# Adverse Events of Acupuncture: A Systematic Review of Case Reports

**DOI:** 10.1155/2013/581203

**Published:** 2013-03-20

**Authors:** Shifen Xu, Lizhen Wang, Emily Cooper, Ming Zhang, Eric Manheimer, Brian Berman, Xueyong Shen, Lixing Lao

**Affiliations:** ^1^Acupuncture Department, Shanghai Municipal Hospital of Traditional Chinese Medicine, Shanghai 200071, China; ^2^Center for Integrative Medicine, School of Medicine, University of Maryland, East Hall, 520 W. Lombard Street, Baltimore, MD 21201, USA; ^3^College of Acupuncture-Moxibustion and Tuina, Shanghai University of Traditional Chinese Medicine, Shanghai 201203, China; ^4^Department of Integrative Medicine, Shanghai Chest Hospital, Shanghai 200030, China

## Abstract

Acupuncture, moxibustion, and cupping, important in traditional Eastern medicine, are increasingly used in the West. Their widening acceptance demands continual safety assessment. This review, a sequel to one our team published 10 years ago, is an evaluation of the frequency and severity of adverse events (AEs) reported for acupuncture, moxibustion, and cupping between 2000 and 2011. Relevant English-language reports in six databases were identified and assessed by two reviewers. During this 12-year period, 117 reports of 308 AEs from 25 countries and regions were associated with acupuncture (294 cases), moxibustion (4 cases), or cupping (10 cases). Country of occurrence, patient's sex and age, and outcome were extracted. Infections, mycobacterial, staphylococcal, and others, were the main complication of acupuncture. In the previous review, we found the main source of infection to be hepatitis, caused by reusable needles. In this review, we found the majority of infections to be bacterial, caused by skin contact at acupoint sites; we found no cases of hepatitis. Although the route of infection had changed, infections were still the major complication of acupuncture. Clearly, guidelines such as Clean Needle Technique must be followed in order to minimize acupuncture AEs.

## 1. Introduction

Traditional acupuncture, which is defined as needling insertion, moxibustion thermal stimulation, and cupping techniques at acupuncture points [1], has become popular in the United States and the rest of the world in recent decades. Data released by the National Institutes of Health (NIH) in 2008 reported that 3.1 million American adults and 150,000 children used acupuncture in 2007. Adult use of acupuncture increased by approximately a million people in the five years from 2002 to 2007 [2]. This increased use brings attention to the safety and quality of the modality. 

A number of large surveys on the safety of acupuncture have been conducted, mainly in Europe. Most reported incidents have been fairly minor, and incidence rates were low. For example, in a prospective survey of 34,000 treatments by traditional acupuncturists, MacPherson et al. [3] found no serious adverse events (AEs) and 43 minor ones, a rate of 1.3 per 1000 treatments. In another prospective survey, Melchart et al. [4] found 7.1% minor AEs and 5 serious ones among 97,733 acupuncture patients. The authors of these studies concluded that serious AEs seem to be rare and that acupuncture is generally a safe intervention.

More than a decade since our last review [5], we have conducted this systematic follow-up review of case reports published between 2000 and 2011 on AEs and complications associated with acupuncture. Our purpose is to (1) estimate the trend of occurrences of the AEs associated with acupuncture over the past 11 years, (2) identify risk factors in acupuncture practice in order to minimize such events, and (3) recommend safe acupuncture practices based on these reported incidents in order to enhance professional standards of practice. 

## 2. Materials and Methods

### 2.1. Search Strategy

We searched six databases in an attempt to locate any and all existing English-language case reports on acupuncture AEs published between 2000 and 2011 in electronic form. PubMed, Medline, the Central Information System of Complementary Medicine (CISCOM), Excerpta Medica (EMBASE), Citations in Nursing and Allied Health Literature (CINAHL), and the Complementary and Alternative Medicine for Pain (CAMPAIN) were searched. Search terms were “acupuncture, acupuncture anesthesia, acupuncture analgesia, electroacupuncture, acupuncture points, auricular acupuncture, moxibustion, needling, and cupping.” These terms were combined with “safe, safety, adverse event, adverse reaction, side effects, complications, and risk.”

### 2.2. Inclusion and Exclusion Criteria

Only original case reports of complications or AEs of acupuncture, moxibustion, and cupping published from 2000 to 2011 were included in this review. Two authors independently screened the titles and abstracts of all papers found from the initial search. Disagreements between the two authors were resolved through discussion.

We excluded multiple inclusions and analyses of the same AE as well as irrelevant studies. An irrelevant study was defined as a non-case report, such as a review, commentary, or clinical trial.

AEs reporting infection, internal organ or tissue injury, and other severe consequences are categorized as “complications,” defined as an added difficulty; a complex state; a disease or accident superimposed upon another without being specifically related. Peripheral or secondary effects such as syncope, nausea, or immune reactions are classified as “adverse reactions” [5].

### 2.3. Data Extraction

**Figure 1:**
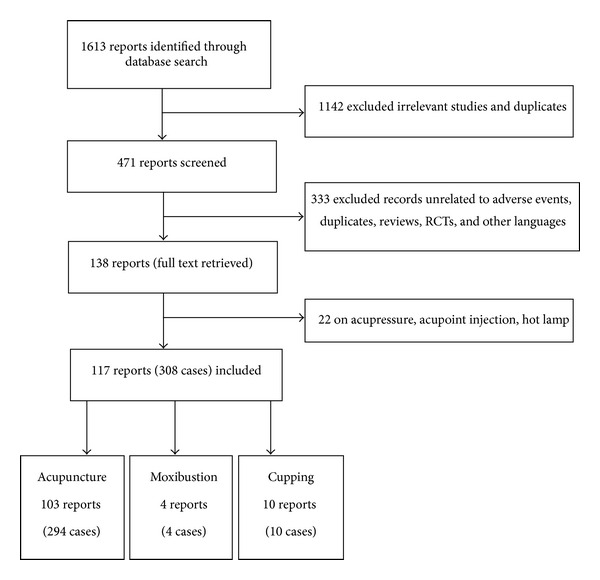
Flow chart of the screening process.

A total of 1613 papers were found; 117 were relevant (Figure 1). When provided, we extracted author, year of publication, country of occurrence, number of patients affected, disease originally treated, preexisting conditions that might have contributed to the AE, the needling site, the reported AE and its outcome, the practitioner's training, and the patient's status at followup. The majority of the reports did not give the date of the AEs. The data were extracted by two independent coauthors, double checked to ensure matching, and organized by whether the AEs were (1) complications or (2) adverse reactions.

## 3. Results

For the years 2000–2011, a total of 117 reports containing 308 AEs associated with acupuncture (294 cases), moxibustion (4 cases), and cupping (10 cases) were identified from 25 countries and regions (Table 1).

### 3.1. Acupuncture Complications: Infections

A total of 239 reported cases were infections associated with acupuncture. These include 48 individual isolated cases reported in 45 papers (Table 2) and 191 cases reported in five outbreaks (Table 3). Incidents were reported in 17 countries and regions. Korea reported 162 cases, Canada 33, Hong Kong 7, Australia 8, Japan 5, Taiwan 5, UK 4, USA 6, Spain 1, Ireland 1, France 1, Malaysia 1, Croatia 1, Scotland 1, Venezuela 1, Brazil 1, and Thailand 1. Most of the papers did not report the practitioner's training, but 4 cases were treated by individuals with no medical training or license [6, 7]. One patient with a knee infection died due to renal failure [8]. All other cases recovered after the infection was treated.

### 3.2. Mycobacterium Infection

Of the 239 cases of infection, 193 (80.75%; 153 from Korea, 32 from Canada, 5 from Hong Kong, 1 from Venezuela, 1 from Brazil, and 1 from Spain) were associated with mycobacterium. 

In 2006, Song et al. reported an outbreak of 40 cases of infection in an Oriental medicine clinic in Republic of Korea. Although disposable acupuncture needles were used, the patients developed skin lesions at two or more sites on the body; infections were confirmed by laboratory culture, clinical signs, and histopathology. All patients recovered after active treatment with antibiotics. Reportedly, these patients received hot-pack therapy and gel massage after acupuncture treatment. No further cases were found in that clinic after equipment sterilization, and regular towel changes were instituted. The authors of the report concluded that the outbreak of infection was due to improper sterilization of equipment applied to the skin after withdrawal of acupuncture needles [52].

In 2006, Tang et al. reported an outbreak of acupuncture-associated bacterial infection in Canada. Between April and December 2002, thirty-two patients developed cutaneous mycobacteriosis after visiting an acupuncture practice in Toronto. Interviews with the patients and acupuncturist revealed that needles were reused and kept in a container of glutaraldehyde disinfectant prior to insertion. The solution was no longer available at the time of the investigation but was probably improperly diluted with tap water [51].

In 2009, Koh et al. reported an outbreak of 109 cases of skin and soft tissue infection in an acupuncture clinic in Republic of Korea. Most patients had at least one skin lesion. Investigators determined that disposable acupuncture needles were used and were unlikely to be the source of infection. Infected patients were all treated by a physical therapy called “interferential current therapy” or “low-frequency therapy.” The authors found that the diluted disinfectant used to sanitize the therapeutic equipment had been prepared several months earlier and was contaminated with *Mycobacterium abscessus*, the likely source of the outbreak [54].

Woo et al. reported four cases of infection by alcohol-resistant mycobacterium, discovered over a two-year period, in patients with skin lesions who were receiving acupuncture treatment in Hong Kong (Table 3). The patients had clinical and/or radiological lesions at acupuncture points. The acupuncturists' training and whether disposable acupuncture needles were used were not reported. The authors recommended that proper infection control guidelines for acupuncture should be mandatory and strictly implemented [50].

### 3.3. Staphylococcus Infection

Nineteen cases from 14 case reports concern staphylococcus infections associated with acupuncture [14, 15, 17, 21, 25, 27, 28, 30, 33, 38, 39, 46, 47, 53]. Of these, nine patients were infected by methicillin-resistant *Staphylococcus aureus* (MRSA): six from Australia [53], one from Korea [33], one from Taiwan [47], and one from Hong Kong [38]. 

In the Australian case, Murray et al. reported a 2008 outbreak of eight cases of invasive MRSA, six of them associated with acupuncture (Table 3). After extensive investigation, the authors concluded that the outbreak most likely resulted from a breakdown in sterile technique during the acupuncture procedure and that the MRSA was probably transmitted from the medical practitioner to the patients. At two time points fifteen months apart, that practitioner had been positively colonized with the MRSA strain that caused the infection [53].

### 3.4. Other Infections

Other infections (31 cases) include septic arthritis [10, 23, 31, 39], necrotizing fasciitis [26, 45, 49], pneumoretroperitoneum [34, 36], facial erysipelas [20], HIV [6], *Listeria monocytogenes*-caused arthritis [31], and infections by *Enterococcus faecalis* [41] and *Pseudomonas *[32, 37]. Although most of the reports did not state possible cause of the infections, reusable needles were used in a few cases.

### 3.5. Acupuncture Complications: Organ and Tissue Injuries

Of 38 cases of organ or tissue injuries, 13 were pneumothoraxes (Table 4); 9 were central nerve system injuries (Table 5); 4 were peripheral nerve injuries (Table 6); 5 were heart injuries (Table 7); 7 were other organ and tissue injuries (Table 8). The cases were distributed among ten countries: 10 from South Korea, 6 from the USA, 6 from Taiwan, 5 from Japan, 3 from the UK, 2 from Germany, 2 from Hong Kong, 1 from Austria, 1 from Iran, 1 from Singapore, and 1 from New Zealand. Although most papers did not report the training background of the practitioner, 3 cases were reportedly treated by individuals with no medical training or license [55–57].

### 3.6. Pneumothorax (Table 4)

Of 13 cases of pneumothorax [58–70] associated with acupuncture, the USA reported 3, the UK 2, Hong Kong 2, Japan 2, Singapore 1, Germany 1, Taiwan 1, and New Zealand 1. Most of these were reported by emergency room physicians. The major patient complaints were dyspnea and chest pain; pneumothorax was confirmed by X-ray. All but one of the 13 patients recovered. A 72-year-old woman died 90 minutes after an acupuncture treatment; autopsy confirmed that the cause was needle penetration of the thoracic cavity [60].

### 3.7. Central Nervous System Injury (Table 5)

There were nine cases of central nervous system injury, including five spinal cord injuries [55, 73–75, 77] and four of brain injury [56, 71, 72, 76]. 

Two of the spinal injuries were caused by migrating broken needles [55, 75]; the others were probably the result of needling too deeply. All patients recovered after treatment.

The brain injuries were an acute intracranial hemorrhage [71], an injury to the medulla oblongata [72], a subarachnoid hemorrhage [76], and an intracranial hemorrhage with cerebellar infarction [56]. Three were due to needle insertion; the medulla injury was caused by a broken needle. Three patients recovered after treatment; outcome was not given for the fourth (Table 5).

### 3.8. Peripheral Nerve Injury (Table 6)

Four reported cases of peripheral nerve injury were associated with acupuncture treatment [78–81], one each in Japan, Korea, the USA, and the UK. The injured nerves were the peroneal nerve via acupuncture point GB34 the median nerve via PC5 and PC6, the facial nerve via ST7 and ST8, and the L5 nerve root via a broken needle in the lumbar region. All patients recovered.

### 3.9. Heart Injury (Table 7)

Five cases of heart injury include two of cardiac tamponade [82, 83], one of the hemopericardium [57], one ventricular embolism [84], and one myocardial injury [85]. Of these, two were due to the migration of embedded needles [83, 84] and two were due to needle insertion [57, 82]. Two were caused by an acupuncturist or TCM practitioner, and one by an “unauthorized acupuncturist” [57]. The status of two practitioners was unreported. Three patients recovered; outcome was not reported in the other two cases.

### 3.10. Other Organ and Tissue Injuries (Table 8)

Seven cases of other organ and tissue injuries were found: a pseudoaneurysm of the abdominal aorta [86], a pseudoaneurysm of the popliteal artery [87], acute traumatic pancreatitis [88], an aortoduodenal fistula causing direct communication between the aorta and the GI tract [89], a rectus sheath hematoma [90], ear hematomas [91], and a popliteal arteriovenous fistula [92]. The patient with acute traumatic pancreatitis had been treated with 13 cm needles placed at three sites on the anterior abdominal wall. Abdominal computed tomography revealed small multiple gold acupuncture needles on the anterior abdominal wall and back muscles. The patient's condition quickly improved with fasting and intravenous fluids [88]. One patient died [89].

### 3.11. Other Complications of Acupuncture

Seven other complications associated with acupuncture were reported (Table 9): bilateral hand edema [93], epithelioid granuloma at needling sites [94], pseudolymphoma [95], localized argyria [96], pustules [97], pancytopenia [98], and scars at needling sites [99]. The localized argyria and pancytopenia were caused by needles embedded 20 and 17 years earlier, respectively [96, 98], in a type of Japanese acupuncture reported in our previous review [5]. The epithelioid granulomas were caused by silicone coating on the needles [94]. The scars were due to a hot needle technique in which the needles were heated in fire before insertion [99].

### 3.12. Adverse Reactions Associated with Acupuncture

Ten cases of adverse reactions from acupuncture were found (Table 10): three of syncope from two reports [100, 101]; two of galactorrhoea (spontaneous milk flow) [102, 103]; one of bilateral nystagmus [104]; one of pyoderma gangrenosum due to immune reaction, in which the tissue became necrotic and deep ulcers formed [105]; one of hepatotoxicity [106]; one of eruptive lichen planus [107]; one of spontaneous needle migration [108]. These unusual cases are uncommonly seen in regular acupuncture practice. The case report authors postulated that these AEs were likely caused by a rare physiological reaction to the acupuncture needle. For example, the case report of spontaneous needle movement involved the acupuncture needles having “spontaneously moved deeper as far as the hilt, travelling an extra depth of 5-10 mm,” which was observed repeatedly on the same patient. Although there was no resulting complicating in this case, the authors cautioned that this could have caused serious complications if the needles had been placed near a vital organ [108].

The syncope cases occurred immediately or several minutes after a first acupuncture treatment; the patients were sitting or semirecumbent during treatment [100, 101].

### 3.13. Complications Associated with Moxibustion

Four AEs associated with moxibustion were found (Table 11): bruising [109], burns and cellulitis [110], spinal epidural abscess [111], and large superficial basal cell carcinoma [112]. Of these, two were self-administered [111, 112]. An “untrained individual” performed the third [110]; there was no information on the fourth [109].

### 3.14. AEs Associated with Cupping

Ten AEs associated with cupping were found (Table 12): four from Turkey, three from Korea, two from Taiwan, and one from the UK. Most were minor: keloid scarring [113], burns [114, 115], and bullae [116, 117]. Several were serious: acquired hemophilia A [118], stroke 14 hours after cupping on the back and neck [119], factitious panniculitis [120], reversible cardiac hypertrophy [121], and iron deficiency anemia [122]. These last two cases involved cupping with bleeding [121, 122]. In six cases, there was no information on practitioner training; in the other four, treatment was self-administered.

## 4. Discussion

Our primary objective in reviewing case reports of AEs associated with acupuncture has been to identify individual cases and outbreaks of AEs and to analyze their possible causes, in order to minimize future acupuncture AEs and enhance safe practice within the profession. How do the objectives and results of this review fit in the context of other available literatures on the safety of acupuncture? Incidence rates for major AEs of acupuncture are best estimated from large prospective surveys of practitioners. Four recent surveys of acupuncture safety among regulated, qualified practitioners, two conducted in Germany [4, 123] and two in the United Kingdom [3, 124], confirm that serious adverse events after acupuncture are uncommon. Indeed, of these surveys, covering more than 3 million acupuncture treatments all together, there were no deaths or permanent disabilities, and all those with AEs fully recovered [125]. Thus, it can be concluded that acupuncture has a very low rate of AEs, when conducted among licensed, qualified practitioners in the West. Recent systematic reviews of RCTs of acupuncture [126–128], in which the acupuncture procedure is also conducted under well-controlled conditions,also found no serious AEs associated with acupuncture [128], although one of these systematic reviews of RCTs separately examined case reports of AEs associated with acupuncture and had findings comparable to ours. However, any medical intervention has the potential to cause damage, particularly when administered by an untrained or unqualified practitioner, or in an unregulated setting. Our objective was thus to identify signals that might suggest the potential for AEs of acupuncture, when administered in specific settings, or when using specific acupuncture styles, and also to compare the patterns of AEs in the past 12 years with the patterns identified in the 35-year period covered by our first review. Comparing the new data with that of the previous review shows the emergence of some important new patterns, which may be relevant for future regulation and policy making.

Although the majority of the AEs are still infections, the routes of infection have changed. Our present findings include 239 AEs from infection; 191 occurred in five outbreaks of bacterial infection caused by skin contact with unsterilized equipment and dirty towels, in unhygienic clinical settings. In our previous findings, hepatitis cross-infections from patient to patient due to reused needles (94 cases reported in four outbreaks) were the most frequent source of infection. Since the introduction of disposable needles, hepatitis infections have rarely been reported, which is an important achievement that has resulted from the greater regulation of acupuncture practice, particularly the requirement for disposable needle use. However, in recent years, bacterial infections, including MRSA and mycobacterium, have become pervasive in healthcare settings in general [129]. Such infections, a pressing concern for all medical practitioners, including acupuncturists, result from poor hygiene. Hygienic clinical settings, sterilized equipment, and clean supplies are critical for preventing future such infections.

Pneumothorax is still the most common organ and tissue injury. There were also cases of spinal cord injuries due to short, small needles embedded laterally along the spine in the Japanese practice known as *okibari*. The putative mechanism responsible for this AE is that the imbedded needles used in the Japanese *okibari* acupuncture technique could spontaneously migrate within the tissue, with some of them migrating to the spinal cord to cause spinal cord injury [130]. However, this AE has significantly decreased since our previous review, in which 11 cases due to this practice were found. In the present review, we found organ injuries mainly to be associated with faulty needle insertion. Heart injuries can be fatal, although no death was reported in the five cases we found. Acupuncture training programs must enhance student knowledge of anatomy at each acupuncture point. Supervised clinical internships must provide rigorous training in needle direction, depth of insertion with attention to the size of the patient, and methods of manipulation.

Three cases reported deaths attributed to acupuncture [8, 60, 89]. Two were due to organ injuries [60, 89], and one was due to infection [8]. Of the organ injury deaths, one case from Japan [60] reported that a 72-year-old woman died after bilateral tension pneumothorax following acupuncture. The finding of the autopsy also suggested the patient that may have been injured by the insertion of the needles into the lungs during the previous acupuncture treatments. The second organ injury death, from Korea, reported that a 68-year-old woman died of massive hematemesis resulting from aortoduodenal fistula. The autopsy showed an injury to the abdominal aorta, caused by a deep insertion with a 15 cm long acupuncture needle into the abdomen [89]. The third case was reported from Scotland in which a 69-year-old man died from an infection after acupuncture treatment at the thigh [8]. The patient was later found to have a preexisting pancytopenia (i.e., low white blood cell count), resulting in an increased susceptibility to infection. The case report author, who is also the practitioner, admitted that the patient's skin at the acupuncture point was not cleaned prior to the needle insertion and later found local muscle infection which led to septicaemia. The patient died a few weeks later from a multiorgan failure. These three unfortunate death cases suggest that biomedical knowledge such as anatomy and microbiology is needed in order avoid organ injury and infection. Skin cleansing should also be required, particularly for those patients with immune compromised condition. 

There were only a handful of cases reported by practitioners who performed the acupuncture [8, 100, 101, 103, 104, 108] including a death report [8]. The rest of the cases were reported by investigators who were not the acupuncturists who performed the treatment. Most cases of AEs did not report the qualification of the practitioner. We would suggest that future report on AEs of acupuncture should include the information on the training qualification of the practitioners and the procedure used for the treatment, such as whether or not clean needle techniques were used.

Acupuncture safety practice guidance or guidelines such as Clean Needle Technique (CNT) appear to have played a critical role in minimizing the number of AEs associated with acupuncture practice [129]. In the United States, CNT was first addressed by the National Certification Commission for Acupuncture and Oriental Medicine in 1984. This course is designed to train professional acupuncturists on safe practice procedures. Course content includes training on microbiology, infection control, skills of adequately setting up a sterile practice area (e.g., adequate use of disinfectant and sterile equipment), adequate needle insertion, and adequate handling of AEs associated with acupuncture [130]. CNT courses are now offered by the US Council of Colleges of Acupuncture and Oriental Medicine and required by the acupuncture licensing boards of each state; as a result, reported acupuncture AE incidents have significantly decreased in the United States. In our previous review, about half of the 202 cases of AE that we identified were from the USA. However, as our present review shows, AE cases reported from the USA are now rare. Of the 308 cases we found, only 13 were from the United States, and out of 239 cases of infection, only 5 are from the United States. It should be noted that there were very few case reports of AEs from China included in this review, although acupuncture is widely practiced in China. We are aware that cases of AEs associated with acupuncture performed in China are likely to be reported in Chinese language case reports, which are not reflected in the present review due to language limitation. We are currently preparing a separate review on AEs reported in China. 

In conclusion, although serious AEs associated with acupuncture are rare, acupuncture practice is not risk-free. Adequate regulation can even further minimize any risk. We recommend that not only adequate training in biomedical knowledge, such as anatomy and microbiology, but also safe and clean practice guidelines are necessary requirements and should continue to be enforced in countries such as the United States where they exist, and that countries without such guidelines should consider developing them in order to minimize acupuncture AEs.

## Figures and Tables

**Table 1:** Adverse events associated with acupuncture, moxibustion, and cupping (2000–2011).

Adverse events	Number of cases
*Acupuncture *	
Complications	284
Infections	239
Isolated incidents	48
Outbreaks	191
Internal organ or tissue injury	38
Pneumothorax	13
Central nerve system	9
Peripheral nerves	4
Heart	5
Other injuries	7
Other complications	7
Adverse reactions	10
*Moxibustion *	4
*Cupping *	10

Total	308

**Table 2:** Infections associated with acupuncture (48 cases).

First author/year (references)	Country	Cases age/sex	Disease treated	Punctured site	Diagnosis	Practitioner	Followup time
Origuchi 2000 [9]	Japan	67/M	Not stated	Not stated	Infectious aortic aneurysm	Not specified	Recovered (≥8 d)
Ishibe 2001 [10]	Japan	13/M	LBP #	Not stated	Septic arthritis	Acupuncturist	Recovered (1 wk)
Woo 2001 [11]	HK	79/F	Knee OA	GB38* (leg)	*Mycobacterium chelonae *	Not specified	Recovered (3 wk)
Nambiar 2001 [12]	UK	42/F	LBP #	Not stated	Endocarditis	Not specified	Recovered (?)
Shah 2002 [13]	UK	37/M	Tendonitis	BL57* (leg)	Streptococcus	Not specified	Recovered (?)
Leavy 2002 [14]	USA	33/M	Hip pain	Low limb	*Staphylococcus aureus *	Not specified	Recovered (6 wk)
Laing 2002 [15]	Ireland	45/F	Postoperative recovery	Around tibia	*Staphylococcus aureus* strain sensitive to methicillin (in knee joint)	Practitioner	Recovered (6 wk)
Uchino 2002 [16]	Japan	47/F	Weight loss	Earlobes	Infected left atrial myxoma (Gram-positive)	Not specified	Recovered (after surgery)
Woo 2003 [17]	HK	73/M	LBP	Back	Staphylococcus	Not specified	Recovered (5 wk)
Ara 2003 [18]	Spain	58/F	Obesity	Abdomen	*Mycobacterium chelonae *	Not specified	Recovered (3 mo)
Cho 2003 [19]	Korea	56/M	Right flank discomfort	Not stated	*Klebsiella pneumoniae *	Not specified	Recovered (?)
Kettaneh 2003 [20]	France	70/F	Not stated	Face	Facial erysipelas	Physiotherapist	Recovered (4 wk)
Wiwanitkit 2003 [6]	Thailand	60/F	muscle pain	Not stated	HIV	Non-MD	Not stated
Ha 2003 [21]	Korea	68/F	LBP	Back	Staphylococcus	Not specified	Recovered (4 mo)
Lin 2003 [22]	Australia	44/F	Not stated	Thigh	Tissue abscess and osteomyelitis	Not specified	Recovered (?)
Daivajna 2004 [23]	UK	48/F	LBP	Low back	Septic arthritis	Not specified	Recovered (3 wk)
Studd 2004 [24]	Australia	64/F	Epigastric pain	Abdomen (embedded needles)	Intra-abdominal abscess	Not specified	Recovered (3 wk)
Kim 2004 [25]	Canada	50/M	LBP	Lower back	Discitis from staphylococcus	Acupuncturist	Recovered (?)
Saw 2004 [26]	Malaysia	55/F	Knee OA	Knee	Necrotizing fasciitis	Not specified	Recovered (?)
Chen 2004 [27]	Taiwan	44/M	Nuchal and subscapular pain	Cervical paraspinal and medial scapular region	*Staphylococcus aureus *	Not specified	Recovered (5 mo)
Vucicevic 2005 [28]	Croatia	53/F	Shoulder stiffness	Shoulder and arm	Staphylococcus pleural empyema	Not specified	Recovered (6 wk)
Bang 2005 [29]	Korea	64/M	LBP	Lumbar paraspinal muscles	*Escherichia coli *	Not specified	Paraplegic
Seeley 2006 [30]	USA	31/M	Hip pain	Bip, thigh	Staphylococcus bacteraemia	TCM doctor	Recovered (4 wk)
Simmons 2006 [8]	Scotland	69/M	Knee pain	SP10* (knee)	Cellulitis, septicemia, and pneumonia	Not stated	Death due to renal failure
Tien 2008 [31]	Taiwan	78/M	Knee RA	Knee	*Listeria monocytogenes* Septic arthritis	Acupuncturist	Recovered (3 wk)
Morgan 2008 [32]	USA	16/F	Weight loss	Auricular	*Pseudomonas aeruginosa *	Acupuncture parlor	Recovered (21 d)
Lee 2008 [33]	Korea	79/M	LBP	Back	*Escherichia coli* and MRSA	Not specified	Recovered (76 d)
Hwang 2008 [34]	Korea	25/F	LBP	Back	Pneumoretroperitoneum	OMD	Recovered (1 wk)
Jeong 2009 [35]	Korea	24/F	Weight loss	Both arms	Factitial panniculitis	Not specified	Recovered (?)
		22/F	Weight loss	Abdomen	Factitial panniculitis	Not specified	Recovered (?)
Hwang 2008 [36]	Korea	25/F	LBP	Not stated	Pneumoretroperitoneum	Licensed OMD	Recovered (7 d)
Wu 2009 [37]	Taiwan	12/M	Neurologic sequelae of encephalitis	Head	Pott's puffy tumor from pseudomonas	Not specified	Recovered (8 wk)
Woo 2009 [38]	HK	43/F	Knee pain	Knee	MRSA	Not specified	Recovered (3 mo)
Ogasawara 2009 [39]	Japan	50/F	LBP	Lower back	Septic arthritis (MRSA)	Not specified	Recovered (70 d)
Guevara-Patiño 2010 [40]	Venezuela	23/F	Not stated	Not stated	NTM	Not specified	Recovered (6 mo)
Nakajima 2010 [41]	Japan	60/F	Knee pain	Needles embedded at knee	*Enterococcus faecalis* knee infection	Not specified	Recovered (1 y)
Winter 2010 [42]	USA	21/F	Obesity	Auricular	Auricular cellulitis	Acupuncturist	Recovered (2 d)
		30/M	Obesity	Auricular	Auricular cellulitis	Acupuncturist	Recovered (1 wk)
Kim 2010 [43]	Korea	53/F	LBP	Lower back	Psoas abscess	Not specified	Recovered (2 wk)
Cho 2010 [44]	Korea	59/F	Not stated	Abdomen, thigh	Mycobacterium skin infection	Not specified	Recovered (3 mo)
		77/M	Not stated	Back and abdomen	Cutaneous tuberculosis infection	Illegal treatment	Recovered (1 y)
Kim 2010 [7]	Korea	72/F	Not stated	Back, shoulder, and right thigh	Cutaneous tuberculosis infection	Illegal treatment	Recovered (9 mo)
		75/F	Not stated	Back and thigh	Cutaneous tuberculosis infection	Illegal treatment	Recovered (9 mo)
Macuha 2010 [45]	USA	84/M	Osteoarthritis	Left groin	Necrotizing fasciitis	Not specified	Recovered (2 mo)
Buckley 2011 [46]	UK	15/M	Eczema	Around the knee	*Staphylococcus aureus* endocarditis	Not specified	Recovered (3 mo)
Kuo 2011 [47]	Taiwan	57/M	LBP	Bilateral paraspinal muscles	MRSA	Not specified	Recovered (2 mo)
Castro-Silva 2011 [48]	Brazil	59/M	Ankle pain	Limb	*Mycobacterium haemophilum* infection	Not specified	Recovered (4 mo)
Hsieh 2011 [49]	Taiwan	44/F	Calf pain	Calf	Necrotizing fasciitis	TCM doctor	Recovered (21 d)

MRSA: methicillin-resistant *Staphylococcus aureus* Infection.

NTM: nontuberculous mycobacterial skin infection.

*Acupuncture points.

**Table 3:** Infectious outbreaks associated with acupuncture (191 cases).

First author/year (references)	Country	Cases	Diagnosis	Practitioner	Followup time
Woo 2002 [50]	HK	4	Alcohol-resistant mycobacteria	Not specified	Recovered
Tang 2006 [51]	Canada	32	Mycobacteriosis	Acupuncturist	Recovered
Song 2006 [52]	Korea	40	Mycobacteriosis	Oriental medical clinic	Recovered
Murray 2008 [53]	Australia	6	MRSA	Acupuncturist	Recovered
Koh 2010 [54]	Korea	109	Mycobacteriosis	Acupuncturist	Recovered

**Table 4:** Pneumothoraxes associated with acupuncture (13 cases).

First author/year (reference)	Country	Cases age/sex	Disease treated	Punctured site	Practitioner	Followup
Kao [58]	Taiwan	28/F	Back pain	Thoracic spine bilaterally	Not specified	Recovered (2 d)
Leung 2002 [59]	HK	70/F	Asthma	Thoracic spine bilaterally	Acupuncturist	Not stated
Iwadate 2003 [60]	Japan	72/F	Stiff neck	Thoracic cavity	Acupuncture clinic	Death
Peuker 2004 [61]	Germany	38/F	Breathing problem	Points at chest and upper back (LU1 and BL13)	Medical acupuncturist	Recovered (1 wk)
Saifeldeen 2004 [62]	UK	31/M	Shoulder pain	Right scapular region	Not specified	Recovered (1 wk)
Lee 2005 [63]	HK	36/F	Back pain	Upper back	Registered TCM practitioner	Recovered (5 d)
Chauffe 2006 [64]	USA	27/M	Upper back pain	Upper back (T2-8 levels)	Not specified	Recovered (2 d)
Su 2007 [65]	Singapore	52/F	Chronic bronchitis	Upper back (T3)	Not specified	Recovered (2 d)
Von Riedenauer 2007 [66]	USA	25/M	Shoulder pain	Migration of embedded needles	Not specified	Recovered (1 wk)
Juss 2008 [67]	UK	50/F	Neck and back pain	Acupoints at upper back (BL13, BL14, BL15, and BL16)	Physiotherapist	Recovered (2 d)
Richter 2008 [68]	New Zealand	35/F	Back pain	Back region	Physiotherapist	Recovered (10 d)
Kennedy 2010 [69]	USA	54/F	Musculoskeletal pain	Left side chest	Not specified	Recovered (?)
Inayama 2011 [70]	Japan	37/F	Not stated	Neck and upper back	Acupuncturist	Recovered (12 d)

**Table 5:** Central nervous system injuries associated with acupuncture (9 cases).

First author/year (reference)	Country	Cases age/sex	Disease treated	Punctured site	Complication	Onset after acupuncture	Practitioner	Followup
Choo 2000 [71]	USA	44/M	Neck pain	GV16 (neck)	Acute intracranial hemorrhage	Immediately	Not specified	Recovered (10 d)
Hama 2004 [72]	Japan	70/M	Not stated	Not stated (broken needle)	Medulla oblongata injury, left facial paresthesia	3 wk	Not specified	Recovered (1 y)
Eftekhar 2005 [73]	Iran	74/M	LBP	Lumbar region	Epidural hematoma	Shortly	Not specified	Recovered (after surgery)
Chen 2006 [74]	Taiwan	30/M	Back pain	Upper back	Epidural haematoma	1 h	Acupuncturist	Recovered (after surgery)
Ulloth 2007 [75]	USA	52/M	LBP	L1, L2, and L3 Vertebrae (embedded needles)	Cerebrospinal fluid fistula	14 mo	Acupuncturist	Recovered (after surgery)
Liou 2007 [55]	Taiwan	29/M	Stiffness of neck	Epidural space at C2 level (a broken needle)	Spinal Cord Injury	3 y	“Nonmedical practitioner”	Recovered (after surgery)
Tsukazaki 2008 [76]	Japan	32/F	Not stated	GV16 (neck)	Subarachnoid hemorrhage	1 d	Oriental medicine clinic	Not stated
Lee 2011 [77]	Korea	58/F	Quadri- paresis neck pain	Neck	Cervical epidural hematoma	1 h	Family physician	Recovered (8 wk)
Heo 2011 [56]	Korea	65/M	Not stated	Posterior neck	Intracranial hemorrhage and cerebellar infarction	3 d	Unauthorized acupuncturist	Recovered (1 mo)

**Table 6:** Peripheral nerve injuries associated with acupuncture (4 cases).

First author/year (reference)	Country	Cases age/sex	Disease treated	Punctured site	Complication	Onset after acupuncture	Practitioner	Followup
Sato 2003 [78]	Japan	62/F	Sciatica	Anterior of the leg	Peroneal nerve palsy	1 d	Not specified	Recovered (4 mo)
Patrick 2005 [79]	USA	63/F	LBP	Low back	Injury of the L5 nerve root	28 y	Not specified	Recovered (after surgery)
Rosted 2007 [80]	UK	47/M	TMD	ST6, ST7 (face)	Bell's Palsy	1 d	Not specified	Recovered (2 wk)
Lee 2008 [81]	Korea	47/M	Abdominal discomfort	PC5 & PC6 (forearm)	Median nerve neuropathy	Shortly	Oriental medicine practitioner	Recovered (1 y)

**Table 7:** Heart injuries associated with acupuncture (5 cases).

First author/year (reference)	Country	Cases age/sex	Disease treated	Punctured site	Complication	Onset after acupuncture	Practitioner	Followup
Kirchgatterer 2000 [82]	Austria	83/F	Not stated	Sternum	Cardiac tamponade	20 min	Experienced acupuncturist	Recovered (2 wk)
Park 2004 [83]	Korea	49/F	Shoulder pain	Shoulders and upper back	Cardiac tamponade	2 h	Not specified	Recovered (after surgery)
Kim 2006 [84]	Korea	70/M	Chronic lung disease	Neck, chest, and abdomen (embed needles)	Right ventricular embolism	1 y	Not specified	Not stated
Song 2010 [85]	Korea	69/F	Pain	Shoulders and neck (implanted needles)	Myocardium injury	10 y	Traditional medicine practitioner	Unknown
Kim 2011 [57]	Korea	54/f	Myalgia and dyspepsia	Chest, abdomen	Hemopericardium	30 min	Unauthorized acupuncturist	Recovered (6 d)

**Table 8:** Other organ or tissue injuries associated with acupuncture (7 cases).

First author/year (reference)	Country	Cases age/sex	Disease treated	Punctured site	Complication	Onset after acupuncture	Practitioner	Followup
Kim 2002 [86]	Korea	54/M	Abdominal pain	Back	Pseudoaneurysm of abdominal aorta	Immediately	OMD	Recovered (8 d)
Kao 2002 [87]	Taiwan	61/F	Osteoarthritis	Knee	Pseudoaneurysm of the popliteal artery	6 mo	Not specified	Recovered (in 1 y)
Uhm 2005 [88]	Korea	42/F	Dyspepsia	Abdomen	Acute traumatic pancreatitis	5 h	Acupuncture clinic	Recovered (4 d)
Chang 2005 [89]	Korea	68/F	LBP	Abdomen	Aortoduodenal fistula	2 wk	Not specified	Dead
Cheng 2005 [90]	Taiwan	37/F	Weight loss	Abdomen	Rectus sheath hematoma	4 h	Not specified	Recovered (1 mo)
Usichenko 2006 [91]	Germany	78/M	Postoperative pain	Ear lobe (embedded needles)	Ear hematomas	4 d	Not specified	Recovered with discoloration
Kuo 2010 [92]	Taiwan	39/F	Knee soreness	Popliteal fossa	Popliteal arteriovenous fistula	Several years	Not specified	Discharged

**Table 9:** Other complications associated with acupuncture (7 cases).

First author/year (reference)	Country	Case age/sex	Disease treated	Puncture site	Complication	Followup time	Remarks
McCartney 2000 [93]	UK	52/M	LPB	LI4 (Hand)	Bilateral hand edema	Recovered (in 8 wk)	No lab evidence of inflammation
Yanagihara 2000 [94]	Japan	55/F	Shoulder pain and lumbago	Back, hip, neck, legs and arms	Epithelioid granuloma at needling sites	Improved	Caused by silicone coating on needles
Kim 2002 [95]	Korea	37/F	Abdominal discomfort	Not state	Pseudolymphoma	Improved	CD-30 positive
Takeishi 2002 [96]	Japan	66/F	Arthralgia	Extremities	Localized argyria	Not stated	Embedded silver needles 20 y earlier
Murray 2002 [97]	UK	35/M	Tennis elbow	Arm	Pustules	Not stated	Pt has Behcet disease
Vassiou 2003 [98]	Greece	67/F	LBP	Chest & abdomen	Pancytopenia	Not stated	Embedded needles 17 y earlier
Pigatto 2004 [99]	Italy	36/F	Hyperthyroidism	St10 (neck)	Scars at needling site	No improvement	“Hot needle” used

**Table 10:** Adverse reactions associated with acupuncture (10 cases).

First author/year (reference)	Country	Case age/sex	Disease treated	Puncture sites	Adverse reactions	Remarks
Castro-Durán 2000 [105]	Spain	48/F	Arthralgia	Not stated	Pyoderma gangrenosum	Immune response
Jenner 2002 [102]	UK	41/F	Cancer pain	Points at upper back	Galactorrhoea	Breast cancer
Cole 2002 [100]	USA	25/M	Healthy volunteer for a clinical study	ST36 (bilateral)	Convulsive syncope	Pt was sitting
Campbell 2005 [103]	UK	32/F	Foot pain	Local points at foot	Galactorrhoea (left side)	Pt had no lactation prior to the tx
Kung 2005 [101]	Taiwan	72/M	Arm pain	LI11, TB5 (arm)	Syncope	Pt was sitting
		63/F	Ankle pain	GB34, B40 (leg & ankle)	Syncope	Pt was sitting
Bradbury 2006 [104]	UK	50/F	Shoulder pain	Points around shoulder	Nystagmus	Semirecumbent position
Smyth 2007 [108]	Scotland	55/M	Back pain	Back	Spontaneous needle movement	No complication
Hong 2008 [106]	China	52/F	Leg weakness	ST36 (leg)	Hepatotoxicity	Pt was in menopause
Fleming 2011 [107]	UK	41/F	Back pain	Lower back	Eruptive lichen planus	Immune response

**Table 11:** Adverse events associated with moxibustion (4 cases).

First author/year (reference)	Country	Case age/sex	Disease treated	Moxibustion site	Adverse events	Practitioner	Remarks
Fisman 2002 [109]	Canada	38/M	Not stated	Abdomen	Ecchymoses	Not specified	Pt had a hx of liver disease
Chau 2006 [110]	USA	53/F	Headache	Leg and feet	Cellulitis	Untrained individual	Recovered
Lee 2008 [111]	Korea	78/F	Pain	Fingers	Infection caused spinal epidural abscess	Self	Pt had diabetes
Yun 2009 [112]	Korea	58/M	Abdominal pain	Abdomen	Basal cell carcinoma	Self	Pt. self-treated for 10 y

**Table 12:** Adverse events associated with cupping (10 cases).

First author/year (reference)	Country	Case age/sex	Disease treated	Cupping site	Adverse events	Practitioner	Remarks
Birol 2005 [113]	Turkey	36/F	Cough	Back	Keloid scar	Not specified	Recovered (several days)
Kose 2006 [114]	Turkey	30/M	Back pain	Back	10% burns at shoulder and back	Self	Recovered (11 d)
Tuncez 2006 [116]	Turkey	57/F	LBP	Low Back	Suction bullae	Not stated	Diabetic; cupping lasted 40 min
Weng 2008 [118]	Taiwan	58/F	Not stated	Thigh	Acquired hemophilia A	Not stated	Improved (1 wk)
Sohn 2008 [121]	Korea	66/F	Pain	Not specified	Reversible cardiac hypertrophy	Self	Bloodletting with cupping >10 y, recovered (3 mo)
Lee 2008 [122]	Korea	39/M	Musculoskeletal pain	Back	Iron deficiency anemia	Not stated	Bloodletting with cupping Pt. fully recovered
Lin 2009 [117]	Taiwan	55/M	Not stated	Back	Bullae	Not stated	Recovered (several wk)
Blunt 2010 [119]	UK	55/M	Not stated	Back and neck	Hemorrhagic stroke (14 h later)	Not stated	May be due to stimulation of baroreceptor, neck area
Kulahci 2011 [115]	Turkey	32/M	Back pain	Back	Burns on back and shoulder	Mother	Recovered
Moon 2011 [120]	Korea	56/F		Neck and shoulder	Factitious panniculitis	Self	Recovered (3 mo)
